# Parental unemployment and educational outcomes in late adolescence: the importance of family cohesion, parental education, and family income in a Norwegian study

**DOI:** 10.1177/14034948241228163

**Published:** 2024-02-21

**Authors:** Kristin Gärtner Askeland, Rebecca Lynn Radlick, Tormod BØe, Mari Hysing, Annette M. La Greca, Sondre Aasen Nilsen

**Affiliations:** 1 Regional Centre for Child and Youth Mental Health and Child Welfare, NORCE Norwegian Research Centre, Bergen, Norway; 2 Division of Health and Social Sciences, NORCE Norwegian Research Centre, Bergen, Norway; 3 Department of Psychosocial Science, Faculty of Psychology, University of Bergen, Bergen, Norway; 4 Department of Psychology, University of Miami, Coral Gables, FL, USA

**Keywords:** Adolescents, parental unemployment, family cohesion, school completion, school grades

## Abstract

**Aims:**

The study aimed to investigate the association between parental unemployment and grade point average and school completion in adolescence, and the importance of family cohesion, parental education, and family income in explaining these associations.

**Methods:**

Data stem from the Norwegian cross-sectional 2012 youth@hordaland-survey including 8437 adolescents (53.4% girls). Information on grade point average, school completion, parental education, and family income were retrieved from the National Education Database. Parental work status and family cohesion were assessed by adolescent self-report.

**Results:**

Adolescents with at least one unemployed parent had lower grade point averages (3.49 compared with 3.92, *P*<0.001) and rates of school completion (71.9% compared with 86.6%, *P*<0.001) compared with adolescents with two working parents. The associations between parental unemployment and both grade point average (b = −0.22, 95% confidence interval −0.32, –0.12) and school completion (odds ratio 0.59, 95% confidence interval 0.46, 0.76) partly attenuated but remained significant when taking family cohesion, parental education, and family income into account. There was a significant interaction between parental unemployment and family cohesion on grade point average, in which the positive association between family cohesion and grade point average was weaker for adolescents with unemployed parents.

**Conclusions:**

**Adolescents with parents outside of the workforce are at higher risk of poorer educational outcomes than peers with working parents. Combined with the positive associations between parental education, family cohesion, family income, and educational outcomes, this underscores the importance of parents for adolescent educational outcomes, and suggests that parents and the family situation should be considered when providing academic support for adolescents who struggle in upper secondary school.**

## Background

Late adolescence is an important developmental period in which educational outcomes, including grades from upper secondary school and school completion, lay the foundation for future education and employment [1]. In Norway, about 22% of adolescents do not finish upper secondary school within 5 years. Non-completion in Norway has been associated with an increased risk of long-term sickness absence [1] and disability pension [2]. The yearly societal cost of school dropout has recently been estimated to be NOK 73b due to lost revenues, public expenses, and lower lifespan expectancy [3]. Educational outcomes are influenced by multiple factors including cognitive abilities [4], mental health [5], social support [6], and school climate [7]. Variables at the family level are also important [8], and are the focus of investigations into the intergenerational transmission of education. One of the variables at the family level that can influence educational outcomes is parental unemployment, and it has been suggested that this is more important in adolescence than earlier in childhood [9]. One explanation for this potential relationship between parental unemployment and educational outcomes is that children observe and learn from their parents, modelling their behaviour and attitudes to, for example working or education, in line with social learning theory [10]. However, it is uncertain whether parental unemployment has an effect beyond other important family variables, such as family cohesion, parental education, and family income.

Parental unemployment has been associated with a variety of measures of school functioning in adolescence, from thoughts and feelings about school and education [11, 12] to grades [9, 13–15], and school completion [16]. More specifically, paternal unemployment had a short term, negative effect on children’s educational aspirations [11], and has also been associated with school burnout [12]. For educational outcomes, which encompass school absence, grades and school completion, parental unemployment has been associated with higher rates of school absence [17], and an increased probability of grade retention [18]. Regarding grades, findings are conflicting. Studies from Finland [9], Denmark [19], and Norway [14] have found small associations between parental unemployment and a lower grade point average (GPA), while a Swedish study only found an association with maternal unemployment [20]. In contrast, a German study did not find an association between parental unemployment and GPA [13]. This could be related to the high workplace participation in Norway [21], both among men and women, contributing to the norm being two working parents. Findings regarding graduation rates are conflicting, in which a study from Germany found a negative association between paternal unemployment and completion of upper secondary school for boys, but not girls [16], while a recent Swedish study did not find an association for either gender [20]. Taken together, the literature suggests a small negative association between parental unemployment and adolescent’s educational outcomes. Nevertheless, there are conflicting findings and the mechanisms behind these associations are less certain. The effect of unemployment on family functioning is often the focus when trying to explain this association; however, few studies have had comprehensive measures of family functioning at their disposal when investigating such questions.

The negative effect of parental unemployment on family functioning has been suggested as an underlying mechanism for its association with academic progress [22]. Reduced family communication and parenting quality are among the possible negative effects of parental unemployment on family functioning [11].

Previous research has shown negative associations between parental involvement and support and school absence and dropout [23, 24]. In the context of parental unemployment, studies have used vastly different measures of family functioning and generally have not found them to explain the association with educational outcomes [11, 13]. A reduction in fathers’ wellbeing and communication with their child had little influence on the association between unemployment and educational aspirations in a British study [11]. In addition, supportive parenting or fathers’ life satisfaction did not mediate their child’s entry into higher education in a German study [13]. Still, these existing studies have used unvalidated and single-item measures of family functioning.

As supportive parent–child relationships have been found to protect against school dropout [24], it is an interesting avenue to utilise more comprehensive measures to investigate further. There is also a lack of studies investigating the importance of family functioning on the association between parental unemployment and grades and school completion, which are important educational outcomes in adolescence.

Parental unemployment is related to a reduction in income, which has been suggested as an important explanatory factor for poorer educational outcomes in children. According to the family stress and the family investment models [25] parental unemployment may trigger economic strain within the family, which in turn may induce parental stress and mental health problems, reducing parents’ capacity to supervise and engage in their adolescents’ academic progress. Related, low income may reduce parents’ financial investments in their children’s academic progress, such as buying books or living in high-income neighbourhoods with better schools. Still, income could not explain the entire association in a longitudinal study [22]. Results vary between studies, and studies from the Nordic countries have generally concluded that income cannot explain the association between parental unemployment and educational outcomes [9, 11, 14]. The relatively lower importance of family income in these countries has been related to their generous social security systems and policies, and free schooling [9]. Indeed, it has been suggested that income is not the main driver of the intergenerational effects of education in Norway, but rather parental education has a larger relative effect than income on their children’s education [26].

The associations between parental unemployment and educational outcomes might differ according to parental education level, but this has received little focus in the literature. Two studies from Finland reported that higher education among parents can compensate for the negative effects of unemployment [9, 12]. In particular, higher parental education seemed to buffer against school burnout for adolescents with unemployed parents [12], and there was no association between unemployment and GPA among adolescents of parents with general secondary or higher education [9]. There is reason to believe that parental education would influence the association between parental unemployment and education in a Norwegian setting, where there is a strong association between parental education and children’s educational outcomes [26].

The sex of the parent and the adolescent could be important moderators of the association between parental unemployment and educational outcomes. Studies have found an effect of paternal, but not maternal education on grade repetition [22] and school performance [14]. There are also indications that the negative impact of parental unemployment is larger for boys [12, 16]. However, a recent study from Finland suggested only minor sex differences in these associations [9], and there is a need for future studies to examine such moderation effects.

### Aims

The main aim of this study was to investigate the association between parental unemployment and the educational outcomes of their adolescent children, measured by GPA and completion of upper secondary school. We also assessed the possible moderating effect of the sex of the non-working parent and the sex of the adolescents on these associations. In addition, we aimed to investigate the importance of family cohesion, parental education, and family income as factors that potentially explain the association between parental unemployment and adolescent educational outcomes.

## Methods

The present study is based on the youth@hordaland-survey of adolescents in Hordaland County in Western Norway, conducted in 2012. All adolescents born from 1993 to 1995 (16–19 years old at the time of the survey) who resided in the county at the time of the survey were invited to participate. Adolescents enrolled at school were sent an invitation to their school email address, while those not in school were sent the invitation by postal mail to their home address.

The main aim of the youth@hordaland-survey was to gain information on adolescent mental health and service use. The questionnaire was comprehensive, covering symptoms of a range of mental health problems, as well as socioeconomic status, relevant lifestyle factors, and protective factors. Questionnaire completion took about 45 minutes and the schools allocated one school hour for participation. School staff were present during data collection to ensure confidentiality, and project team members were available on telephone throughout the data collection period.

### Sample

Of the invited adolescents, 10,253 consented to participate and filled out the questionnaire. Of these, 9166 adolescents consented to linkage to registry information. The present study includes the adolescents who consented to registry linkage and responded to the questions regarding parental work status. Parental work status was coded as missing for adolescents who reported their parents to be retired (mothers *n*=4, fathers *n*=58) or deceased (mothers *n*=33, fathers *n*=51). There were 326 adolescents with missing data on both the parental work status variables who were removed from the sample. In addition, 403 adolescents who reported their parents to be on sick leave or disability pension were removed, yielding a total sample of 8437 adolescents.

### Ethics

The youth@hordaland-survey and the linkage to the National Education Database (NUDB) were approved by the Regional Committee for Medical and Health Research Ethics in Western Norway and the Norwegian Agency for Shared Services in Education and Research. A data protection impact assessment was conducted for the linkage in compliance with the general data protection regulation. According to Norwegian regulations, adolescents 16 years of age and older can make decisions regarding their own health, including participation in health studies. Thus, while parents/guardians received written information about the study in advance, the adolescents gave consent to participate.

### Instruments

#### Demographic factors

Sex and age were derived from the participants’ personal identification number. Age was calculated by subtracting the date of participation from the date of birth.

#### Parental unemployment

The adolescents were asked about their parents’ occupations in separate open-ended questions for mothers and fathers. Their answers were coded according to the ISCO-08 classification. Parents were defined as working if they had a work ISCO code or were reported to be students. Parents were defined as unemployed if they were reported not to work, including unemployed parents, or homemakers (parents staying at home without employment). There was no information available on the duration of the unemployment in the present study. In addition to the separate categories for maternal and paternal unemployment, a combined variable of parental unemployment was created, indicating whether one or both parents were unemployed.

#### Information from the NUDB

##### Grade point average

The adolescents’ GPA for the school year 2011/2012 (the year of the data collection) was retrieved from the NUDB. The grades in upper secondary education range from 1 (failure) to 6 (excellent), and the GPA is the mean of all the grades a student received in one school year.

##### School completion

Completion of upper secondary school was defined according to the definition of dropout used by Statistics Norway. Dropout is defined as not having received a diploma within 5 years of starting upper secondary for students in general tracks, and within 6 years for students in vocational tracks.

##### Parental education level

The NUDB contains information on the highest education level of both parents when the respondent was 16 years old. The variable was categorised into: ‘lower’ (completed lower secondary education), ‘upper secondary’ (completed upper secondary education), and ‘higher’ (completed college or university education). A combined measure of parents’ educational level was created, indicating the highest completed education in the family by either the mother or the father.

##### Household income

Information about household income in 2011 (after tax) was obtained from the NUDB. Household income was rescaled using the EU equivalence scale to be comparable across households of different compositions and with different numbers of working adults.

#### Family cohesion

Family cohesion was measured by a subscale in the resilience scale for adolescents (READ) [27]. We used the family cohesion subscale identified in a psychometric evaluation of the READ in the youth@hordaland-sample [28], which includes one more item than the original version of the subscale (‘In my family we have rules that simplify everyday life’). This item was originally placed in the subscale structured style [27]. The modified family cohesion subscale consisted of the average of seven statements rated on a five-point Likert scale ranging from ‘totally disagree’ (1) to ‘totally agree’ (5). The subscale measures support and shared values in the family (e.g. ‘In my family we support each other’, ‘I feel comfortable with my family’).

### Statistical analyses

Differences between adolescents with parents in and outside the workforce regarding GPA and school completion were investigated using the independent samples *t*-test and chi-square test, respectively. To ease the interpretation of the results, our measures of GPA, family cohesion, and family income were standardised (*z*-transformed) prior to the regression analyses, with a mean of 0 and a standard deviation (SD) of 1.

The associations between maternal unemployment, paternal unemployment and the combined measure of parental unemployment and GPA were investigated in separate linear regression analyses. Model 1 included unemployment and the sex of the adolescent. Model 2 added an interaction term between unemployment and adolescent sex. The same models were tested with school completion as the outcome, using logistic regression.

The importance of parental education was investigated in separate analyses for maternal, paternal, and combined parental education for GPA and school completion. In model 1, parental unemployment was included as a predictor of GPA in a linear regression analysis, and as a predictor of school completion in a logistic regression analysis. Maternal, paternal, and combined parental education were entered in model 2 in separate analyses, and model 3 also included an interaction between maternal/paternal/parental education and parental unemployment. There was no evidence of interactions with the educational level of the parents (see Supplemental Table II).

The associations between different family variables and GPA were tested using linear regression. In the crude model, associations between parental unemployment, family cohesion, parental education, family income, and GPA were investigated separately. Parental unemployment was included as a predictor of GPA alongside family cohesion in model 1, parental education in model 2, and family income in model 3. In model 4, all variables were included. The same models were tested with school completion as the outcome, using logistic regression.

The analyses were conducted using Stata, version 17.

## Results

Of the total sample, 449 adolescents reported that one or both of their parents were unemployed. Parents outside the workforce generally had lower educational qualifications and lower income than working parents (see Table I for details).

**Table I.** Demographic characteristics.

	Both parents working		Parental unemployment	
	*n*=7988 (94.7%)		*n*=449 (5.3%)	
	*n*	%	*n*	%
Sex				
Girl	4266	53.4	240	53.5
Age (m (SD))	16.9	0.85	16.9	0.84
Ethnicity: from Norway				
Mother	7371	92.4	361	80.8
Father	7256	91.2	354	80.0
Maternal education				
Lower	1147	14.5	146	35.1
Upper secondary	3299	41.7	180	43.3
Higher	3459	43.8	90	21.6
Paternal education				
Lower	1122	14.4	106	25.1
Upper secondary	3868	49.7	203	48.1
Higher	2791	35.9	113	26.8
Income (m (SD))^a^	3.53	1.58	2.82	2.02
Family cohesion (m (SD))	3.90	0.82	3.72	0.94

SD: standard deviation.

a Equivalent household income in NOK 100,000 (about €8560).

Adolescents with one or more parents outside the workforce had a lower GPA (mean 3.49, SD 0.06) compared with adolescents with two working parents (mean 3.92, SD 0.01, *t*(83993) = 8.40, *P*<0.001, two-tailed). Furthermore, a lower percentage of adolescents with parents absent from work had completed upper secondary education within 5 years (71.9% compared with 86.6%, χ^2^(1, *N*=8430) = 76.04, *P*<0.001).

When investigating maternal and paternal unemployment separately and combined, only minor differences between the estimates were observed, both for GPA and school completion (see Supplemen-tal Table I). There were no significant interactions between the sex of the adolescent or parent and any of the variables regarding unemployment.

When investigating maternal and paternal education separately and combined, there were no clear differences between the estimates, both for GPA and school completion (see Supplemental Table II). Lower parental education was negatively associated with GPA and school completion, while higher parental education was positively associated with GPA and school completion across analyses.

**Figure 1.**
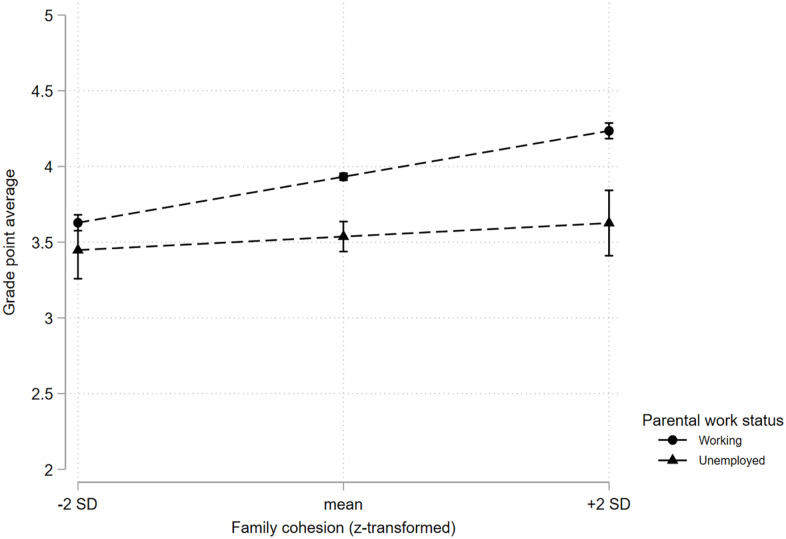
The association between parental unemployment and grade point average (GPA) at different levels of family cohesion.

**Figure 2.**
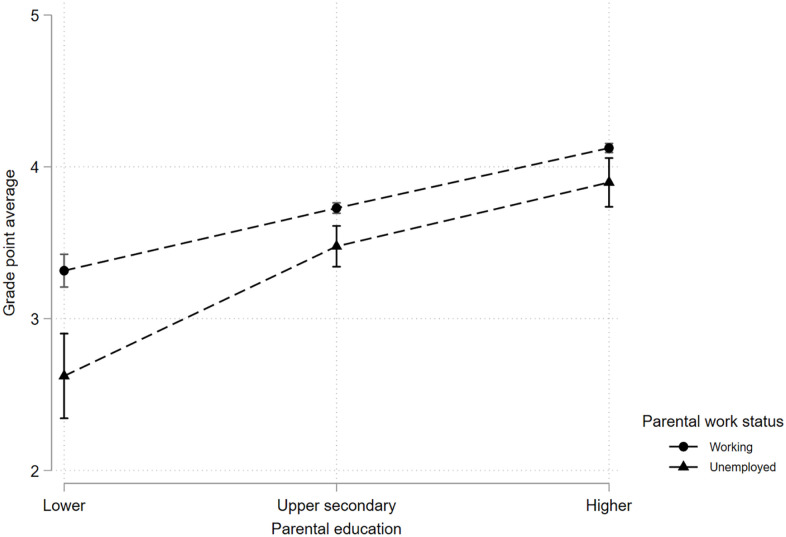
The association between parental unemployment and grade point average (GPA) at different levels of parental education.

Parental unemployment, family cohesion, parental education, and family income were all significantly associated with GPA when investigated separately (see Table II). Adjusting for family cohesion, parental education, and family income in separate analyses reduced the strength of the association between parental unemployment and GPA (from b = −0.43 to b = −0.38 (family cohesion), b = −0.29 (parental education), and b = −36 (family income)). When all predictors were entered in the full model, the association between parental unemployment and GPA was still significant but reduced to b = −0.22 (95% confidence interval (CI) –0.32, –0.12). Adding parental ethnicity reduced the association to b = −0.21 (95% CI −0.31, –0.10). There was a significant interaction between parental unemployment and family cohesion on GPA, in which the positive association between family cohesion and GPA was weaker for adolescents with unemployed parents (see Figure 1). There was further a significant interaction effect between parental unemployment and parental education on GPA, in which the negative association between parental unemployment and GPA was larger for adolescents with parents with low education, compared with upper secondary and higher education (see Figure 2). There was no significant interaction effect between family income and parental unemployment on GPA.

**Table II.** The importance of family cohesion, parental education, and family income on the association between parental unemployment and grade point average.

	GPA									
	Crude		Model 1		Model 2		Model 3		Model 4	
	coef	CI	coef	CI	coef	CI	coef	CI	coef	CI
Parental unemployment	**–0.43**	–0.52, –0.33	**–0.38**	–0.48, –0.28	**–0.29**	–0.39, –0.19	**–0.36**	–0.46, –0.26	**–0.22**	–0.32, –0.12
Family cohesion	**0.15**	0.13, 0.17	**0.14**	0.11, 0.17					**0.13**	0.11, 0.15
Parental education^a^										
Lower	**–0.49**	–0.59, –0.38			**–0.47**	–0.57, –0.36			**–0.47**	–0.58, –0.36
Higher	**0.40**	0.36, 0.45			**0.40**	0.35, 0.44			**0.34**	0.30, 0.39
Family income	**0.14**	0.12, 0.16					**0.13**	0.11, 0.15	**0.07**	0.05, 0.09

Significant associations (at *P*<0.05) are indicated in bold type.

GPA: grade point average; CI: confidence interval.

Family cohesion and family income are standardised, and the coefficients correspond to changes in GPA following a one standard deviation increase from the mean in each of the measures.

a Upper secondary education.

Similarly, parental unemployment, family cohesion, parental education, and family income were all significantly associated with school completion in the crude model (see Table III). Adjusting for family cohesion, parental education, and family income in separate analyses reduced the association between parental unemployment and school completion (from odds ratio (OR) 0.39 to OR 0.42, OR 0.46, and OR 0.51, respectively). The association was further reduced but remained significant in the full model (parental unemployment OR 0.59, 95% CI 0.46, 0.76). Adding parental ethnicity reduced the association to OR 0.60, 95% CI 0.47, 0.77. There were no significant interaction effects between any of the included variables and parental unemployment on school completion.

**Table III.** The importance of family cohesion, parental education, and family income on the association between parental unemployment and school completion.

	School completion									
	Crude		Model 1		Model 2		Model 3		Model 4	
	OR	CI	OR	CI	OR	CI	OR	CI	OR	CI
Parental unemployment	**0.39**	0.32, 0.49	**0.42**	0.34, 0.53	**0.46**	0.37, 0.58	**0.51**	0.41, 0.64	**0.59**	0.46, 0.76
Family cohesion	**1.36**	1.28, 1.44	**1.34**	1.27, 1.43					**1.31**	1.23, 1.40
Parental education^a^										
Lower	**0.40**	0.32, 0.50			**0.42**	0.34, 0.53			**0.43**	0.34, 0.54
Higher	**2.26**	1.97, 2.59			**2.21**	1.93, 2.53			**1.93**	1.67, 2.23
Family income	**1.83**	1.66, 2.02					**1.74**	1.58, 1.92	**1.47**	1.32, 1.63

Significant associations (at *P*<0.05) are indicated in bold type.

OR: odds ratio; CI: confidence interval.

Family cohesion and family income are standardised, and the coefficients correspond to changes in school completion following a one standard deviation increase from the mean in each of the measures.

a Upper secondary education.

## Discussion

Adolescents with at least one parent outside the workforce had lower grades and lower rates of school completion compared with adolescents with two working parents. These associations were similar when considering maternal and paternal unemployment separately and combined, and for boys and girls. The associations between parental unemployment and GPA and school completion were partly explained by family cohesion, parental education, and family income. Parental education had the observed strongest attenuating effect on the association between parental unemployment and GPA. For school completion, the attenuating effects were strongest and similar for parental education and family cohesion. Higher levels of family cohesion were related to greater increases in GPA for adolescents whose parents both worked. Furthermore, the negative effect of parental unemployment on GPA was larger when the parents had only completed lower education.

Our finding that parental unemployment is a risk factor for poor educational outcomes in adolescence is in line with the existing literature [9, 11, 12, 17, 18], and theoretical expectations [10]. More specifically, our finding that the mean GPA was lower among adolescents whose parents were not working is comparable with previous Finnish [9], Danish [19], and Norwegian [14] studies, but in contrast to a German study [13]. Regarding school completion, our findings are in line with a study from Germany [16], and are also consistent with studies showing reduced educational aspirations [11] and a higher occurrence of school burnout [12], which could be precursors to secondary school dropout. Of note, these findings are in contrast to a recent Swedish study in which parental job loss was not associated with high school completion [20].

We did not find evidence for parent–gender-specific associations. The associations were similar for maternal and paternal unemployment, in contrast to some previous studies suggesting paternal unemployment to be more important [13, 14, 22]. This discrepancy could be due to the high workplace participation among Norwegian women, and high gender equality, both in the workplace and in the homes [21]. Indeed, a recent study from Sweden, with similar gender equality as Norway, found an association for maternal and not paternal unemployment [20]. Along these lines, a study from Germany found that fathers’ unemployment was associated with higher educational attainment among girls, which the authors suggest could be due to conservative gender roles, and an attempt to seek qualifications to find a spouse with higher education and thus lower risk of unemployment [16]. They further note that it would be interesting to investigate this in more egalitarian societies [21], and in the Norwegian context we did not find evidence of such gender differences.

Greater family cohesion was associated with higher GPA and school completion, in line with previous studies of family cohesion and school absenteeism and dropout in adolescence [23, 24]. Furthermore, family cohesion explained part of the association between parental unemployment and the educational variables, and the potential benefits of higher family cohesion scores on GPA were smaller for adolescents whose parents were not working. This contrasts with previous studies in which various measures of family functioning did not mediate or moderate the association between parental unemployment and educational outcomes [11, 13]. Notably, the educational outcomes are not directly comparable, as the previous studies have investigated associations with schooling ambitions [11] and entry into tertiary education [13]. This finding could be understood in the context of social learning theory [10], in which adolescents who have a close relationship with their parents may be more likely to model their behaviours after their parents. Thus, the positive effect of family cohesion on educational outcomes is stronger when the parents model behaviours and attitudes related to work participation.

Parental education level explained part of the association between parental unemployment and adolescent educational outcomes. There was a significant interaction effect between parental education and parental unemployment on GPA, but not school completion. The finding regarding GPA is in line with studies from Finland where higher parental education compensated for the negative effect of parental unemployment on school burnout, GPA, and secondary education [9, 12]. Similarly, Stevens and Schaller [18] found a larger effect of job loss on grade repetition when the parents had lower education. This could also be understood in the context of social learning theory [10], in which one can assume that highly educated parents model positive attitudes towards education to their children.

Family income explained part of the association between paternal unemployment and both GPA and dropout, consistent with some studies [22, 29], but contrary to studies conducted in England [14] and the Nordic countries [9, 11]. This is somewhat surprising as these discrepancies have previously been explained by the social welfare policies in the Nordic countries. Still, it is important to note that family income was associated with rather small reductions in the associations between parental unemployment and both GPA and school completion, and the influence of family income was reduced when the other family variables were included. The discrepancy with previous Nordic studies could be related to the different measures of parental unemployment, in which previous studies have focused on more immediate effects of job loss [11], and have not included long periods of unemployment and the related long lasting negative economic consequences [9]. In the present study we did not have information on the duration of the unemployment and also included home makers, who are likely to be unemployed for an extended time period. Indeed, insufficient income and fear of lacking money have previously been associated with ‘inadequate’ parenting and subsequent reduced adolescent academic achievement [30].

### Strengths and limitations

A key strength of the study is the use of high-quality registry data on GPA and school completion combined with a self-report measure of family cohesion with multiple items from a validated resilience scale that has been thoroughly psychometrically evaluated on the current sample [28]. Furthermore, we used objective register-based measures of family income and parental education level that are devoid of the limitations common to self-reported measures.

A central limitation is the measure of parental unemployment which was based on adolescent self-report. There was no information available on the duration of the unemployment, which could be important in the context of parental unemployment [13]. Given the cross-sectional design of the study, the temporal order cannot be established. For instance, for some adolescents with chronic illness, their illness could influence parental work–life participation over time. We do not have longitudinal information on both parental unemployment and GPA, which could help untangle the association further. Furthermore, we did not have data on other variables that could be important, such as which schools the adolescents attended. However, this had little effect on estimates from a previous register-based study from Norway [14].

Finally, attrition from the study may have impacted the generalisability of the results. Some adolescents may have dropped out of school before the study period, and thus are not included in the current sample. However, the mean GPA of the study sample was identical to the mean GPA of Norway in 2012, which lends support to the representativeness of the sample regarding one of our main dependent variables [31].

## Conclusions and implications

Adolescents with parents outside the workforce are at increased risk of school dropout and lower grades in comparison with adolescents with two working parents. This risk indicator may help to identify groups who could benefit from preventive interventions. Parental and family support may be one viable strategy due to the importance of family-level factors for these associations [32].

## Supplemental Material

sj-docx-1-sjp-10.1177_14034948241228163 – Supplemental material for Parental unemployment and educational outcomes in late adolescence: the importance of family cohesion, parental education, and family income in a Norwegian study Supplemental material, sj-docx-1-sjp-10.1177_14034948241228163 for Parental unemployment and educational outcomes in late adolescence: the importance of family cohesion, parental education, and family income in a Norwegian study by Kristin Gärtner Askeland, Rebecca Lynn Radlick, Tormod BØe, Mari Hysing, Annette M. La Greca and Sondre Aasen Nilsen in Scandinavian Journal of Public Health
