# Supplementary material for: Parental unemployment and educational outcomes in late adolescence: the importance of family cohesion, parental education, and family income in a Norwegian study
Source: Scand J Public Health. 2024 Feb 21;53(1):52–61. doi: 10.1177/14034948241228163 (PMC11742708; doi:10.1177/14034948241228163)
Supplement: sj-docx-1-sjp-10.1177_14034948241228163 – Supplemental material for Parental unemployment and educational outcomes in late adolescence: the importance of family cohesion, parental education, and family income in a Norwegian study [file sj-docx-1-sjp-10.1177_14034948241228163.docx]

Table S1. The association between parental unemployment and GPA and school completion.

|  | GPA | | | | School completion | | | |
| --- | --- | --- | --- | --- | --- | --- | --- | --- |
|  | Model 1 | | Model 2 | | Model 1 | | Model 2 | |
|  | coef | CI | coef | CI | OR | CI | OR | CI |
| Maternal unemployment | **-0.47** | -0.58, -0.35 | **-0.51** | -0.67, -0.36 | **0.41** | 0.32, 0.53 | **0.39** | 0.28, 0.56 |
| Maternal unemployment *sex |  |  | 0.10 | -0.12, 0.33 |  |  | 1.09 | 0.67, 1.79 |
| Paternal unemployment | **-0.53** | -0.71, -0.35 | **-0.48** | -0.71, -0.25 | **0.28** | 0.20, 0.40 | **0.28** | 0.17, 0.45 |
| Paternal unemployment *sex |  |  | -0.13 | -0.48, 0.23 |  |  | 1.02 | 0.49, 2.09 |
| Maternal and/or paternal unemployment | **-0.47** | -0.56, -0.37 | **-0.46** | -0.60, -0.33 | **0.39** | 0.32, 0.49 | **0.36** | 0.27, 0.49 |
| Maternal and/or paternal unemployment *sex |  |  | 0.08 | -0.12, 0.28 |  |  | 1.18 | 0.47, 1.83 |

Note. Significant associations are indicated in bold. GPA: grade point average, CI: confidence interval, OR: odds ratio.

Table S2. The importance of parental education for the association between parental unemployment and GPA and school completion.

|  | GPA | | | | School completion | | | |
| --- | --- | --- | --- | --- | --- | --- | --- | --- |
|  | Model 1 | | Model 2 | | Model 1 | | Model 2 | |
|  | coef | CI | coef | CI | OR | CI | OR | CI |
| Parental unemployment | **-0.43** | -0.52, -0.33 | **-0.26** | -0.36, -0.16 | **0.39** | 0.32, 0.49 | **0.49** | 0.39, 0.62 |
| Maternal education^1^ |  |  |  |  |  |  |  |  |
| Lower |  |  | **-0.34** | -0.40, -0.27 |  |  | **0.51** | 0.44, 0.60 |
| Higher |  |  | **0.34** | 0.30, 0.39 |  |  | **1.86** | 1.60, 2.16 |
|  |  |  |  |  |  |  |  |  |
| Parental unemployment | **-0.43** | -0.52, -0.33 | **-0.34** | -0.43, -0.24 | **0.39** | 0.32, 0.49 | **0.43** | 0.34, 0.54 |
| Paternal education^1^ |  |  |  |  |  |  |  |  |
| Lower |  |  | **-0.30** | -0.37, -0.24 |  |  | **0.55** | 0.47, 0.64 |
| Higher |  |  | **0.36** | 0.31, 0.41 |  |  | **2.10** | 1.78, 2.47 |
|  |  |  |  |  |  |  |  |  |
| Parental unemployment | **-0.43** | -0.52, -0.33 | **-0.29** | -0.39, -0.19 | **0.39** | 0.32, 0.49 | **0.46** | 0.37, 0.58 |
| Parental education^1^ |  |  |  |  |  |  |  |  |
| Lower |  |  | **-0.47** | -0.57, -0.36 |  |  | **0.42** | 0.34, 0.53 |
| Higher |  |  | **0.40** | 0.35, 0.44 |  |  | **2.21** | 1.93, 2.53 |

Note. Significant associations are indicated in bold. GPA: grade point average, CI: confidence interval, OR: odds ratio.

^1^Ref: Upper secondary education
